# Reviewing 75 years of the WHO: successes, challenges and opportunities

**DOI:** 10.5588/pha.23.0029

**Published:** 2023-06-21

**Authors:** G. N. Kazi

**Affiliations:** Editor-in-Chief, *Public Health Action*, The Union

Belated congratulations to the WHO, which was created on 7 April 1948 and has now reached its Diamond Jubilee. The anniversary fell on World Health Day, with the theme of ‘Health For All’, which has been the WHO’s focus since ‘the attainment by all people of the highest possible standards of health’ was written into its constitution. The WHO has been the United Nations leading agency for global health and currently has over 7,000 staff members at its headquarters in Geneva, Switzerland, six regional offices (catering to the differing health needs of each region) and 150 country offices providing technical support to its 194 Member States. During its 75-year history, it has achieved singular successes, such as launching the primary healthcare (PHC) approach in 1978, the eradication of small pox in 1979, creation of the Stop TB Partnership and responding to disease outbreaks, most notably, the COVID-19 pandemic. The theme of ‘Health For All’ is particularly relevant at this crucial time – there are less than 7 years left to attain Universal Health Coverage and other targets encompassed in Sustainable Development Goal (SDG) 3 (including the elimination of TB, AIDS, malaria and diabetes). The WHO coordinates international health issues, advises governments on developing public health and social services, supports governments in eradicating disease, formulates standards for medical education and scientific research, and is mandated as the directing and co-ordinating authority on international health.

The world has dramatically changed since the First World Health Assembly met in Geneva in 1948 and established malaria, TB, venereal diseases, maternal and child health, sanitary engineering, and nutrition as its priorities.^1^ The WHO has a strong normative role to play, and many low- to middle-income countries are heavily dependent on its technical and scientific support to fulfill their obligations on the right to health. Many years after the Alma Ata Declaration in 2005, the World Health Assembly ratified a resolution demanding that every person should be able to access health services and not be subjected to financial hardship. The Union has the role of a non-State actor in official relations with the WHO: ‘official relations’ is granted to non-governmental organisations that contribute significantly to the advancement of public health, and have a sustained and systematic engagement in the interest of the WHO. The Union has collaborated with the WHO on several issues, such as prioritising TB care during COVID-19, following up on fulfilling pledges made at the 2018 United Nations High-Level Meeting on TB, air pollution, access to medicines and vaccines, antimicrobial resistance, health of refugees and migrants, new diagnostic tools and investing in research and development.^2^

Dr Halfdan Mahler was a highly effective Director General of WHO for three terms (1973–1988); during which time the PHC approach gained international recognition. Thirty years later in 2008, he recalled how, at the end of the Alma Ata Conference in 1978, a young African woman physician in beautiful African dress read out the Conference Declaration while many people had tears in their eyes. He commented:

Health for all is a value system with primary health care as the strategic component. The two go together. Years later, WHO continues to implement the same consensus with diverse results in different regions and countries. Primary healthcare is more urgently needed now than ever before.

He further clarified that in 1978,

…the goal was not to eradicate all diseases and illnesses by 2000; we knew that would have been impossible. Our goal was to focus world attention on health inequities and attain an acceptable level of health, equitably distributed throughout the world.

Dr. Mahler astutely pointed out that the success of primary healthcare hinges on our ability to foster participation from individuals, families, and communities. However, it is crucial to recognise that community involvement alone cannot thrive without the necessary support from the healthcare system.^3^ With hindsight, it appears that two vital elements of the PHC methodology were consistently underplayed – inter-sectoral action and involvement of communities and the private sector in health, which led to sup-optimal results (even though such partnerships already existed in WHO’s Civil Society Initiative and as proposals developed for the Health for All Initiative). Currently, there is a growing need to pay greater attention to marginalised groups, traditionally excluded from the political agenda-setting and policy-making. Private sector, community and civil society involvement have been explicitly mentioned in the SDGs and the UN document, *Transforming our World and the 2030 Agenda for Sustainable Development,* which recognise the positive role of such partnerships. Nevertheless, a concerning reality persists in several countries, wherein there is either an absence of government policy regarding the role of the private health sector or a lack of tangible plans to effectively execute such public policies. Consequently, an urgent need arises to address this void and rectify the situation promptly.^4^

Here the COVID-19 pandemic also merits discussion. The success of the Sustainable Development Goals (SDGs) has been contended to be contingent upon sustained economic growth and global integration. However, the unprecedented impact of COVID-19 has inflicted severe damage on both fronts. Consequently, it has become imperative to reexamine the SDGs in terms of their prioritisation, developmental capacity, and resilience in light of the significant disruptions faced globally.^5^ The pandemic has exerted enormous strain on all countries, exposing gaps in public health and exacerbating inequities, necessitating the alignment of varied priorities in strengthening health systems.^6^ Meanwhile, the WHO also needs a degree of organisational introspection. Its response to the Ebola crisis provided dramatic evidence of its limited operational competence.^7^ It also highlighted problems, such as an overstaffed bureaucracy and poorly organised decentralised structures, which impaired the work of the regional offices. Furthermore in 2023, the removal of the Regional Director of the WHO’s West Pacific Region for abusive behaviour was highly unsettling,^8^ while it also has to contend with the political ramifications of the COVID-19 pandemic.^9^ To deliver on the SDG agenda, the WHO will need to focus on its neutral provision of technical assistance to countries by harnessing the best experts and centres of excellence, swiftly declaring public health emergencies and urgently responding to pandemic threats. It should also adhere to international health regulations and reach out to the World Bank and other development partners, for increased development support for poorer countries, while improving its effectiveness by enhancing its regular budget through greater domestic spending by member countries.^10^

For its part, the international community needs to establish an equitable financing mechanism to ensure a fair burden among the rich and poor countries. This, in turn, will enable WHO to address the political and social determinants of health by providing institutional mechanisms leading to social equity and the prerequisite of health as an inalienable human right. Meanwhile, we congratulate WHO on its anniversary and wish it success in all its endeavours.
